# Correlation between Physical Properties of 12-Hydroxystearic Acid Organogels and Hansen Solubility Parameters

**DOI:** 10.3390/gels9040314

**Published:** 2023-04-07

**Authors:** Yuya Murakami, Taisei Uchiyama, Atsushi Shono

**Affiliations:** 1 Department of Industrial Chemistry, Faculty of Engineering, Tokyo University of Science, 6-3-1, Nijuku, Katsushika-ku, Tokyo 125-8585, Japan; 2 Institute of Innovative Research, Tokyo Institute of Technology, 2-12-1, Ookayama, Meguro-ku, Tokyo 152-8550, Japan

**Keywords:** Hansen solubility parameters, low-molecular-weight gelator, 12-hydroxystearic acid, organogel, rheology

## Abstract

The Hansen solubility parameter (HSP) is a useful index for reasoning the gelation behavior of low-molecular-weight gelators (LMWGs). However, the conventional HSP-based methods only “classify” solvents that can and cannot form gels and require many trials to achieve this. For engineering purposes, quantitative estimation of gel properties using the HSP is highly desired. In this study, we measured critical gelation concentrations based on three distinct definitions, mechanical strength, and light transmittance of organogels prepared with 12-hydroxystearic acid (12HSA) and correlated them with the HSP of solvents. The results demonstrated that the mechanical strength, in particular, strongly correlated with the distance of 12HSA and solvent in the HSP space. Additionally, the results indicated that the constant volume-based concentration should be used when comparing the properties of organogels to a different solvent. These findings are helpful in efficiently determining the gelation sphere of new LMWGs in HSP space and contribute to designing organogels with tunable physical properties.

## 1. Introduction

Supramolecular gels fabricated with low-molecular-weight gelators (LMWGs) have a stimulus-responsible nature because they are formed via relatively weak non-covalent bonds between LMWGs [1]. Unlike polymer gels, in which the network made of polymer chains entraps solvents, LMWG molecules stack together and form fibers, and these fibers form self-assembled fibrillar networks (SAFiNs) in supramolecular gels. For this reason, supramolecular gels easily change their state by slight external stimuli, such as temperature, pH, and light [2]. Due to this characteristic, many applications of supramolecular gels have been proposed, including drug delivery [3,4,5], fuel cells [6,7], electromechanical sensors [8], 3D printing [9,10], semi-conductor [11], and artificial muscles [12,13]. 12-hydroxystearic acid (12HSA) is one of the LMWGs that can form supramolecular gels at significantly low concentrations. 12HSA is composed of a long fatty chain with a hydroxyl group on it. The detailed gelation mechanism has been investigated by many researchers, and it has been revealed that 12HSA molecules stack together and form SAFiNs during gelation [14]. The usefulness of 12HSA has been recognized because of its superior gelation ability for a wide variety of solvents [15]. Additionally, 12HSA has high biocompatibility because it can be derived from biodegradable material [14]. For these reasons, it has been commercially used in numerous fields [16].

To extend their versatility, many have attempted to classify solvents that can and cannot be gelated by LMWGs [17,18,19,20]. As a result, the Hansen solubility parameter (HSP) was found to be successful over others [21,22,23,24,25,26,27,28]. Gao et al. identified the solution/gelation and gelation/precipitation boundaries in HSP space for 12HSA and empirically defined solution and gelation spheres in HSP space [22]. The HSP is useful for solvent classification for gelation because the interaction between the solvent and 12HSA is essential in gelation. When the interaction between the two compounds is strong (close distance in HSP space), 12HSA is completely dissolved in the solvent, and gel does not form. On the other hand, if the interaction between the two compounds is too weak, 12HSA may not dissolve in the solvent and thus cannot be used for gelation. In other words, the interaction between 12HSA and the solvent must be of an appropriate magnitude for gelation to occur. The same trend can be seen for other LMWGs, such as 1,3:2,4-dibenzylidene sorbitol and cholesteryl 4-(2-anthryloxy)butanoate [23].

Conventional classification of solvents in HSP space is based on three (or four) classes: solution, (transparent and opaque) gel, and precipitation. However, these classes are not consistent for different concentrations of LMWGs, as shown by Diehn et al. [29]. Therefore, it is necessary to re-classify solvents when the concentration of LMWGs is altered. Moreover, many gels must be prepared to determine the gelation and solution sphere when using this method (Diehn et al. examined 34 solvents for each concentration), which is unsuitable for practical application. From the engineering perspective, the model predicting the minimum LMWG concentration for gelation (critical gelation concentration) or the physical properties of obtained gels is highly valuable. In the past, Rogers et al. attempted to correlate critical gelation concentration (CGC) with various parameters of solvents and demonstrated solid correlations between CGC and hydrogen-bonding HSP [22,24]. Conversely, few attempts have been made to correlate multiple gel properties with HSP. Therefore, even though we suspect the gel can be obtained with a given solvent, we do not know what properties the obtained gel would have with a given LMWG concentration.

In this study, we evaluated organogel properties in terms of three different CGCs (based on weight, molar amount, and volume), mechanical strength, and optical properties, and investigated the correlation between these properties and HSP. Through this investigation, we revealed which organogel properties are strongly correlated with HSP and proposed a method to reduce the number of trials to determine the gelation/precipitation boundary. The results suggested that the properties of organogels can be predicted using the distance in HSP space.

## 2. Results and Discussion

### 2.1. Properties of 12HSA Organogels

Conventionally, the weight fraction of LMGWs has been used to represent LMGW concentration in most research, which is preferable for practical usage. In this work, the following three distinct definitions of 12HSA concentrations were adopted when analyzing the results:

(1) $$w=\frac{W_{12\mathrm{HSA}}}{W_{\mathrm{solvent}}+W_{12\mathrm{HAS}}},$$

(2) $$v=\frac{n_{12\mathrm{HSA}}}{W_{\mathrm{solvent}}/\rho_{\mathrm{solvent}}},$$

and

(3) $$x=\frac{n_{12\mathrm{HSA}}}{n_{\mathrm{solvent}}+n_{12\mathrm{HSA}}}.$$

In Equation (1), $W_{12\mathrm{HSA}}$ and $W_{\mathrm{solvent}}$ [g] denote the weight of 12HSA and solvent in organogels, respectively. Therefore, w [wt%] is equivalent to the weight fraction. In Equations (2) and (3), $n_{12\mathrm{HSA}}$ and $n_{\mathrm{solvent}}$ [mol] denote the amount of 12HSA and solvent molecules in organogels, respectively, and $\rho_{\mathrm{solvent}}$ [g/L] denotes the density of pure solvent used for the organogel preparation. Therefore, v [mol/L-solvent] represents the amount of 12HSA molecules in the unit volume of the pure solvent. The density of pure solvent, not organogel itself, was used here because measuring the density of fragile supramolecular gel is technically difficult. x [mol%] represents molar fraction of 12HSA in organogels. The solvents used to prepare organogels are listed in Table 1, with their HSP. HSP values were obtained from the software Hansen Solubility Parameter in Practice [30].

To determine CGC, organogels were prepared with various concentrations of solvent. After the preparation, the container was inverted and the liquid was separated from the organogel. The degree of gelation was evaluated by the following equation:

(4) $$p_{\mathrm{gel}}=1-\frac{W_{\mathrm{res}}}{W_{\mathrm{tot}}}.$$

In Equation (4), $W_{\mathrm{res}}$ [g] is the weight of separated liquid and $W_{\mathrm{tot}}$ [g] is the total weight of the mixture before separating the liquid. Therefore, $p_{\mathrm{gel}}$ indicates the weight fraction of gel in the whole fluid. When the mixture loses fluidity, $p_{\mathrm{gel}}$ is close to 1.0 since no liquid can be separated from the mixture. In this work, the mixture is considered an organogel when $p_{\mathrm{gel}}>0.98$.

**Figure 1:**
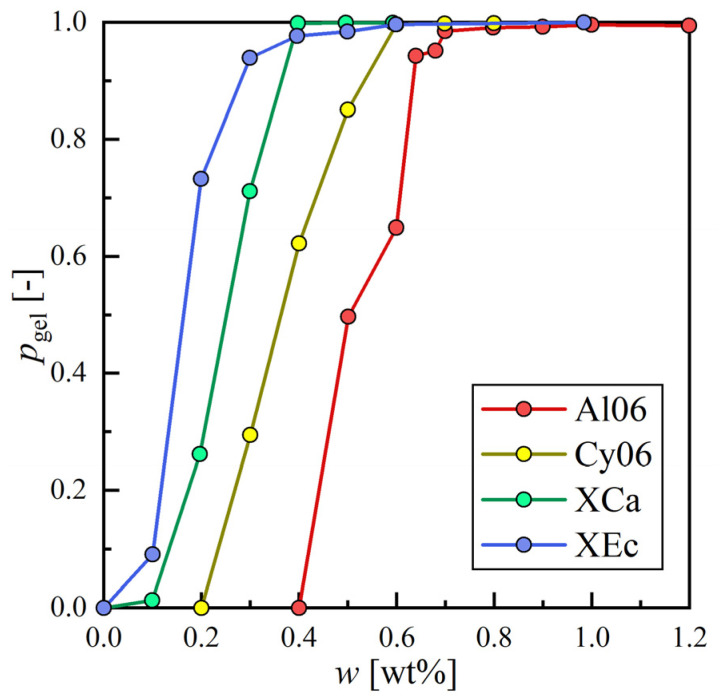
The degree of gelation with a weight fraction of 12HSA for hexane (Al06), cyclohexene (Cy06), hexachloroacetone (XCa), and ethyl chloroformate (Xec).

The changes in $p_{\mathrm{gel}}$ along with *w* were summarized in Figure 1 for four solvents. Gelation progressed with increasing w for all solvents and $p_{\mathrm{gel}}$ approached 1.0. When w is above the solubility of 12HSA in the solution at room temperature, it recrystallizes in the solvent while cooling the mixture. At this point, 12HSA forms self-assembled fibrillar networks (SAFiNs), and if it spans the whole fluid with sufficient durability, the fluid can be regarded as an organogel, which we assume happens when $p_{\mathrm{gel}}>0.98$. The minimum concentration of 12HSA achieving $p_{\mathrm{gel}}>0.98$ is regarded as the CGC of the solvent. Since three distinct definitions were used in this research, CGC varies based on the definition. CGCs based on w, v, and x were defined as $w_{\mathrm{CGC}}$ [wt%], $v_{\mathrm{CGC}}$ [mmol/L-solvent], and $x_{\mathrm{CGC}}$ [mol%], respectively.

The CGCs for each solvent are summarized in Table 1. Additionally, the appearance of typical gels is shown in Figure S1. As Gao et al. reported, a decreasing trend of $w_{\mathrm{CGC}}$ with increasing chain length was observed for alkenes with carbon chain lengths from 7 to 10 [22]. In this study, the values of $w_{\mathrm{CGC}}$ were not in agreement with their results. For example, $w_{\mathrm{CGC}}$ for alkenes with chain lengths from 6 to 10 were 0.2 to 0.4 wt% in their work, while they were 0.7 to 0.9 wt% in this study. The difference may be due to the relatively large volume of the organogels and containers used in this study (25.0 mL and 50.0 mL, respectively), which required more 12HSA to support their weight. The result suggests that although the CGC based on a fluidity test can capture the gelation trend, it is impossible to obtain reproducible results without unifying measurement conditions (e.g., sample volume, container shape, dissolution temperature). Therefore, quantitative evaluation using a standardized method is necessary to evaluate the organogel with high reproducibility.

**Figure 2:**
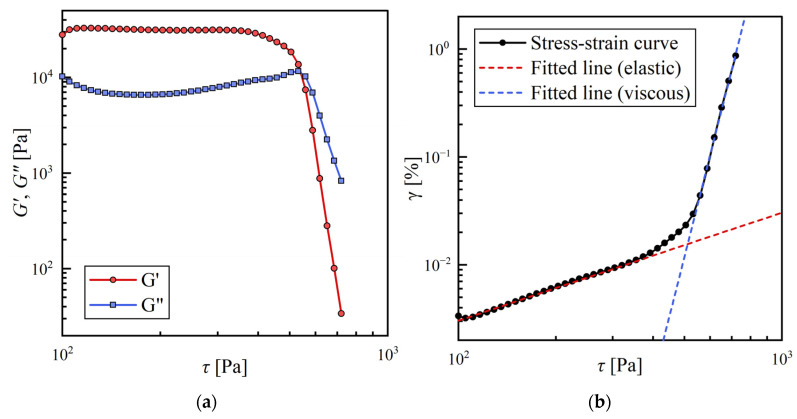
The result of oscillation stress sweep measurements of a 12HSA organogel prepared with styrene (*w* = 1.10%): (**a**) Storage and loss moduli with various stress; (**b**) Stress/strain curve.

**Figure 3:**
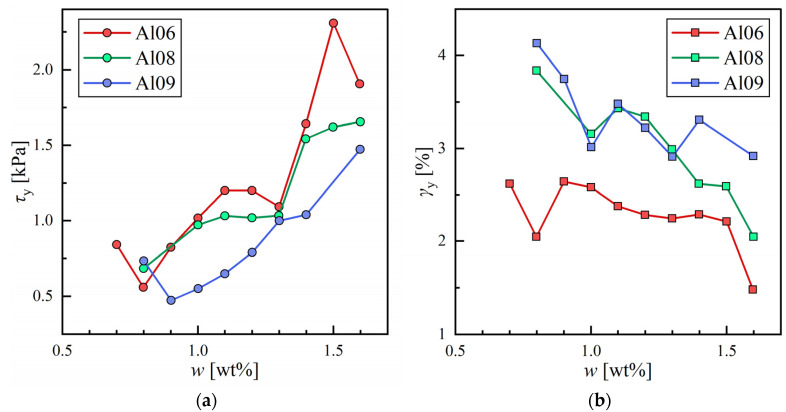
The obtained rheological property of 12HSA organogels prepared with hexane (Al06), octane (Al08), and nonane (Al09): (**a**) Yield stress ($\tau_{y}$) with a weight fraction of 12HSA; (**b**) Yield strain ($\gamma_{y}$) with a weight fraction of 12HSA.

Therefore, in addition to CGC measurements, we conducted viscoelasticity measurements using a rheometer (oscillation stress sweep) to evaluate and compare the mechanical strength of the organogels quantitatively. The complete description of the measurements is given in Materials and Methods. The measurements are shown in Figure 2a. Comparing the storage modulus $G^{\prime }$ [MPa] and loss modulus $G^{\prime \prime }$ [MPa] for the material with $p_{\mathrm{gel}}>0.98$ with given stress (τ), it was found that the relation *G′* > *G″* is always satisfied in the low shear stress region (τ~100 Pa). The result quantitatively confirms that the material is in a gel state (elasticity-dominated). The same trend could be seen for all gelated samples (Figures S2 and S3). Additionally, the yielding of organogels, where $G^{\prime }$ and $G^{\prime \prime }$ decreased rapidly with increasing τ, was observed for all samples. This drastic change is attributed to the fact that the organogel can no longer withstand stress, and the structure collapses at this point. The relationship between τ and strain (γ) in this measurement is shown in Figure 2b. In the elasticity-dominated region, the relationship between these two variables is a straight line with slope 1 in the log scale because γ is proportional to τ. However, when elasticity is lost, this relationship no longer withstands, and a sudden change in slope can be observed. In this study, the yield point is defined as the intersecting point of the fitted line with slope 1 in the elasticity-dominated region and the fitted line in the viscosity-dominated region. τ and γ at the yield point are defined as yield stress ($\tau_{y}$) and yield strain ($\gamma_{y}$). $\tau_{y}\;$and $\gamma_{y}$ along with w for the three alkenes are summarized in Figure 3. For all solvents, $\tau_{y}$ increased and $\gamma_{y}$ decreased as w increased. This change is due to the increased density or strength of SAFiNs made of 12HSA molecules with higher w. Densely packed SAFiNs are difficult to be broken (thus, high $\tau_{y}$) and deformed (thus, low $\gamma_{y}$).

In addition to the mechanical strength, the light transmittance of the organogels was measured. Gao et al. reported that the transparency of LMWG-based organogels varies depending on the type of solvent used; thus, they categorized organogels into opaque and transparent ones [22]. In this study, the transparency of the organogel was quantitatively evaluated from the amount of light transmitted through the organogel using a light-emitting diode and an optical sensor. The transparency ($\alpha_{\mathrm{gel}}$) was defined with the following equation:

(5) $$\alpha_{\mathrm{gel}}=\frac{I_{\mathrm{gel}}}{I_{\mathrm{air}}}.$$

In Equation (5), $I_{\mathrm{air}}$ and $I_{\mathrm{gel}}$ denote the intensity of light transmitted through air and organogel, respectively, measured by the optical sensor. The complete description of the measurement is given in Materials and Methods. With smaller $\alpha_{\mathrm{gel}}$, more light is scattered inside SAFiNs, and the organogel is completely opaque when $\alpha_{\mathrm{gel}}=0.0$.

**Figure 4:**
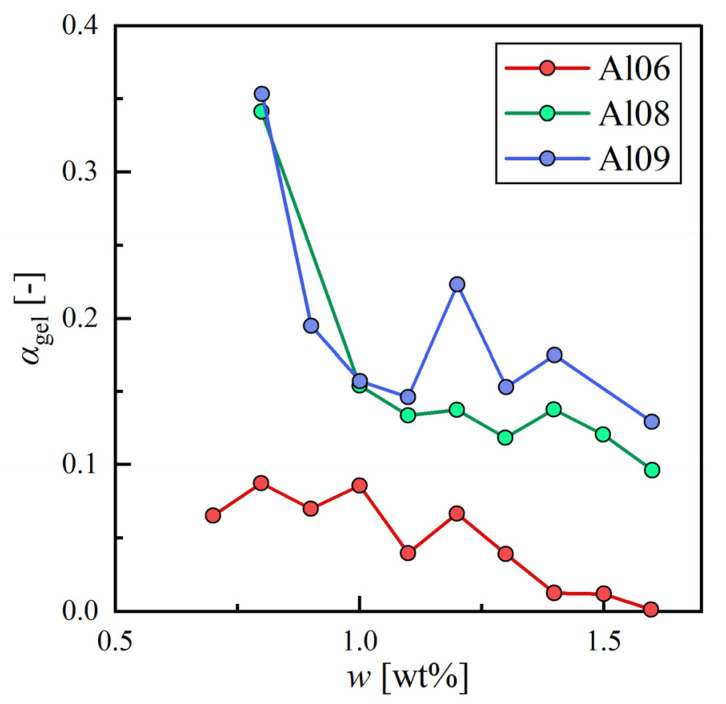
The degree of gelation with a weight fraction of 12HSA for hexane (Al06), octane (Al08), and nonane (Al09).

Figure 4 summarizes the relationship between w and $\alpha_{\mathrm{gel}}$ for the three alkenes. As can be seen, $\alpha_{\mathrm{gel}}$ tended to decrease with increasing w. This trend is caused by the increasing density of SAFiNs in the organogel. Densely packed SAFiNs scatter light more than sparsely packed ones and, therefore, $\alpha_{\mathrm{gel}}$ decreases. Compared to the mechanical strength measurement, the light intensity measurement is inexpensive and quick and, thus, has potential usefulness as a simple quantitative evaluation method for the physical properties of organogels.

### 2.2. Correlation with Hansen Solubility Parameter

In previous works, a correlation between solvents that can be gelated with 12HSA and HSP has been investigated [22,23,24]. However, the quantitative correlation between HSP and the physical properties of the organogels is vague to date. In this study, we quantitatively correlated the HSP with the physical properties of 12HSA organogels in terms of CGC, $\tau_{y}$, and $\alpha_{\mathrm{gel}}$ which were measured as explained in the previous subsection.

HSP is an empirical parameter that represents each molecule with three parameters to evaluate the affinity between two compounds: a dispersion term ($\delta_{D}$), a polarity term ($\delta_{P}$), and a hydrogen bonding term ($\delta_{H}$). In general, the affinity between two compounds is represented by the weighted L2 norm of each molecular parameter ($D^{i,j}$) as follows:

(6) $$D^{i,j}=\sqrt{{\left(2\delta_{D}^{i}-2\delta_{D}^{j}\right)}^{2}+{\left(\delta_{P}^{i}-\delta_{P}^{j}\right)}^{2}+{\left(\delta_{H}^{i}-\delta_{H}^{j}\right)}^{2}}.$$

In Equation (6), superscripts i and j denote two compounds to be evaluated. It is known that the smaller $D^{i,j}$ is, the more similar their molecular structures are; therefore, these compounds tend to be miscible [31]. The parameters of 12HSA were estimated by the group contribution method and the following values were obtained: $\delta_{D}^{12\mathrm{HSA}}=16.6\;\mathrm{MPa}$, $\delta_{P}^{12\mathrm{HSA}}=2.86\;\mathrm{MPa}$, and $\delta_{H}^{12\mathrm{HSA}}=6.77\;\mathrm{MPa}$ [30]. In the gelation with LMWGs such as 12HSA, the dissolution of the LMWG into solvent by heating and the recrystallization of the LMWG by cooling play essential roles. Specifically, the solubility change caused by temperature is a crucial factor in initializing gelation. Therefore, it is not surprising that HSP and gelation behavior have a strong correlation and, in fact, numerous studies have pointed out the high degree of correlation [21,22,23,24,25,26,27,28].

**Figure 5:**
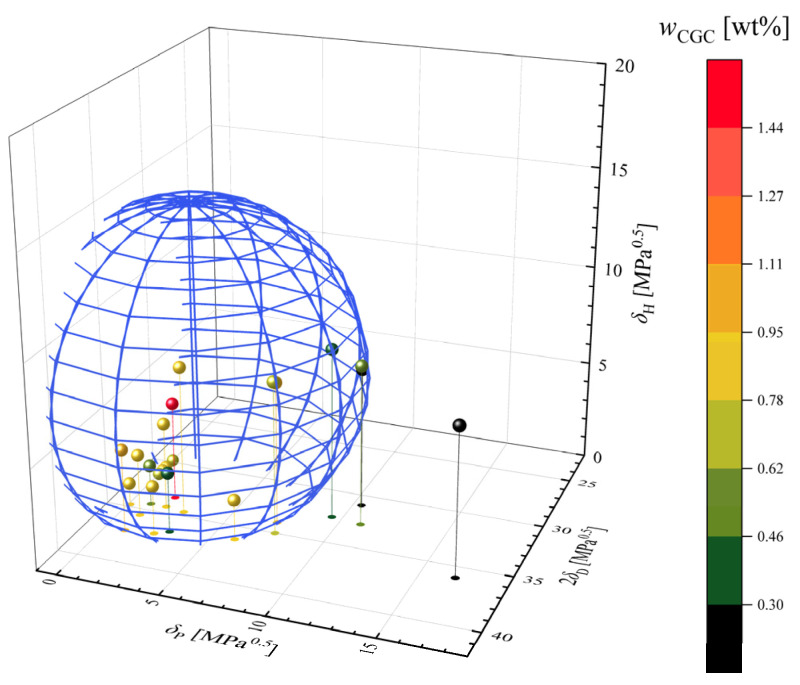
3D plots of solvent used for 12HSA [30] organogel preparation with their $w_{\mathrm{CGC}}$. Note that black plots indicate that 12HSA was insoluble in the solvent.

The relationship between $w_{\mathrm{CGC}}$ and HSP of solvents is summarized in Figure 5. The blue sphere shows the boundary: $D=8.2\;{\mathrm{MPa}}^{0.5}$. Note that D denotes the weighted L2 norm [Equation (6)] between 12HSA and solvent in the HSP space (i.e., $D:=D^{12\mathrm{HSA},\mathrm{solvent}}$). Plots in red indicate solvents with large $w_{\mathrm{CGC}}$ and plots in green indicate solvents with small $w_{\mathrm{CGC}}$. Plots in black indicate that 12HSA is insoluble in the solvents. All gelled solvents (20 solvents in total) satisfied $D<8.2\;{\mathrm{MPa}}^{0.5}$. On the other hand, Acetone, γ-Butyrolactone, and Water, which did not dissolve 12HSA, satisfied $D\geq 7.9\;{\mathrm{MPa}}^{0.5}$. Based on the results, gelation/precipitation boundary should be set at approximately $D=8.0\;{\mathrm{MPa}}^{0.5}$.

Additionally, three different CGCs ($w_{\mathrm{CGC}}$, $v_{\mathrm{CGC}}$, $x_{\mathrm{CGC}}$) were correlated with D. To evaluate the correlation, we calculated the correlation coefficient ($R^{2}$) between the two variables. $R^{2}$ indicates the correlation between two valuables; if $R^{2}$ is close to 1, it suggests that CGC and D have a strong correlation. Additionally, the following normalized error (Δ) is used to evaluate the accuracy of the prediction under an assumption of linear correlation between the two variables:

(7) $$\Delta =\frac{1}{N}\overset{N}{\underset{i}{\sum }}\frac{\left|y_{i}^{\exp}-y_{i}^{\mathrm{pred}}\right|}{\sigma \left(y^{\exp}\right)}.$$

In Equation (7), N denotes the total number of experimental data, $y_{i}^{\exp}$ and $y_{i}^{\mathrm{pred}}$ denote experimental value and calculated value by the linear approximation, respectively, and $\sigma \left(y^{\exp}\right)$ denotes the standard deviation of the experimental value. Since the distribution of the experimental value differs when the definition of CGC differs, the error was normalization by $\sigma \left(y^{\exp}\right)$. By this normalization, we can evaluate the magnitude of the estimation error relative to the distribution width. The results are summarized in Table 2.

For all three CGCs, we obtained $R^{2}<0.27$ and Δ>0.60, suggesting no correlation between D and CGCs. Gao et al. reported that $w_{\mathrm{CGC}}$ and $\delta_{H}^{12\mathrm{HSA}}$ have a strong positive correlation in the range of $3.0\;{\mathrm{MPa}}^{0.5}<\delta_{H}^{12\mathrm{HSA}}<5.0\;{\mathrm{MPa}}^{0.5}$ [22]. However, the aromatic compounds and halides used in this work did not exhibit the same trend. Interestingly, if only alkene and aromatic compounds are used, $v_{\mathrm{CGC}}$ and D have a weak correlation ($R^{2}=0.65$), while $w_{\mathrm{CGC}}$ and $x_{\mathrm{CGC}}$ are still poorly correlated with *D* ($R^{2}<0.27$). Rogers et al. reported that the polymorphic forms of 12HSA are different in polar and apolar solvents [25]. Since the alkane and aromatic compounds used in this work have weak polarity ($\delta_{P}<2.4\;{\mathrm{MPa}}^{0.5}$), and the halides have high polarity ($3.0\;{\mathrm{MPa}}^{0.5}<\delta_{P}<11.0\;{\mathrm{MPa}}^{0.5}$), the 12HSA may form different types of fibers in these solvents. Therefore, it is suggested that polar and apolar solvents should be examined by different correlation models because 12HSA behaves differently in these two types of solvents.

The correlation between HSP and organogel properties was also evaluated regarding mechanical strength. The mechanical strength of organogel was evaluated using $\tau_{y}$. However, since $\tau_{y}$ is a function of 12HSA concentration, as we discussed previously, comparisons between different solvents must be made with a fixed concentration. Moreover, the definition of the concentration must also be fixed to compare different solvents properly. For this reason, we obtained three different values of $\tau_{y}$ at the following three conditions: w=1.0 wt%, v=80 mmol/L−solvent, x=2.8 mol%. The results were summarized in Table 3, Table 4 and Table 5. Note that we used interpolation to calculate $\tau_{y}$ if $\tau_{y}$ was not measured at exactly these concentrations. It should also be noted that the number of the organogel obtained was different if different definitions of concentration were used.

**Figure 6:**
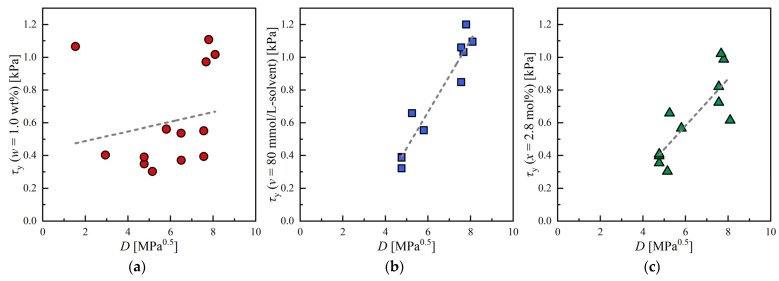
Correlation between yield stress ($\tau_{y}$) and distance in HSP space: (**a**) Fixed concentration at w=1.0%; (**b**) Fixed concentration at v=80 mmol/L-solvent; (**c**) Fixed concentration at x=2.8 mol%.

The correlations between $\tau_{y}$ measured by rheometer and D are given in Figure 6 for three conditions, and values of $R^{2}$ and Δ are listed in Table 2. The results show a strong positive correlation ($R^{2}=0.917$ and Δ=0.218) between D and $\tau_{y}$ measured at v=0.080 mol/L−solvent [see Figure 6b.] In general, the solubility of 12HSA increases as D decreases. This change leads to a decrease in 12HSA molecules used to form SAFiNs at room temperature, as well as the strength of SAFiNs. Although the properties of SAFiNs cannot be measured directly, they should be strongly correlated with the bulk properties of the gels because the loss of fluidity during the gelation is attributed to the formation of SAFiNs [14]. For these reasons, the mechanical strength of organogels increased when 12HSA and solvent were far apart in the HSP space. Intriguingly, such a strong correlation was observed only when v is fixed; $R^{2}$ was as low as 0.037 and 0.673 when w and x were fixed, respectively.

The reason why the correlation could be confirmed only when v is fixed is related to the organogel formation mechanism. During organogel formation using LMWGs, SAFiN spans the entire organogel. In order for SAFiN to spread throughout the organogel and acquire the strength to withstand its weight, a sufficient amount of LMWGs per volume should be added. For solvents with different densities or molar masses, the amount of LMWG per volume is not constant, even if w and x are fixed. On the other hand, v is an excellent indicator to evaluate the amount of LMWG per volume. Therefore, it suggested that when comparing the properties of supramolecular gels between different solvents, comparisons should be made with a fixed v, rather than w, which was frequently used in past reports.

**Figure 7:**
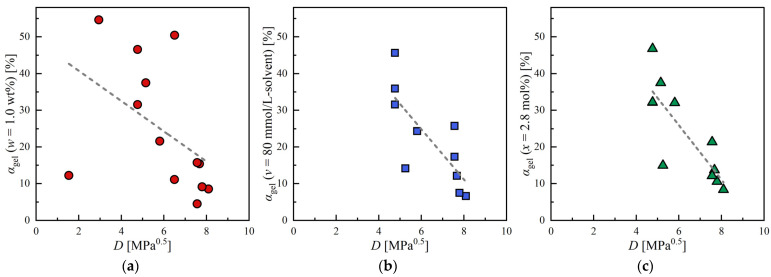
Correlation between light transmittance ($\alpha_{\mathrm{gel}}$) with the distance in HSP space: (**a**) Fixed concentration at w=1.0%; (**b**) Fixed concentration at v=80 mmol/L-solvent; (**c**) Fixed concentration at x=2.8 mol%.

Lastly, $\alpha_{\mathrm{gel}}$ measured by light transmission was evaluated at the three different concentrations (w=1.0 wt%, v=80 mmol/L−solvent, x=2.8 mol%). The results were summarized in Table 3, Table 4 and Table 5. The correlation results between $\alpha_{\mathrm{gel}}$ and D are given in Figure 7, and $R^{2}$ and Δ were listed in Table 2. Weak negative correlations were obtained for $\alpha_{\mathrm{gel}}$ when v or x was fixed. The reason is that the increase in D (decrease in the affinity between 12HSA and solvent) resulted in increasing the amount of 12HSA used to form SAFiN, which leads to scattering of light. On the other hand, the correlation between $\alpha_{\mathrm{gel}}$ and D was not as strong as the correlation between $\tau_{y}$ and D due to the poor reproducibility of the measurement caused by the inhomogeneity of the organogel. However, this measurement is more concise than the rheological measurement and is, therefore, considered a more practical method for evaluating supramolecular gel.

From the above discussion, we found a correlation between HSP and properties of 12HSA organogels using the volume-based concentration (v), instead of the frequently used weight-based concentration (w). In particular, there is a strong correlation between the mechanical strength of the organogel ($\tau_{y}$) and the distance in HSP space (D). This finding will aid in the design of organogels with tunable strength. In addition, CGC and light permeability of organogels also correlate with D; these findings are useful in determining the gelation/precipitation boundary in HSP space. For example, when CGC is large, or the transparency of the organogel is high, the HSP of the solvent is close to that of LMWG; therefore, gelation is likely to occur in solvents with similar HSP. On the other hand, for solvents showing the opposite characteristics, their HSP is close to the limit of the gelation boundary. By incorporating these insights into the conventional HSP-based method, determining the solution and gelation sphere for a new LMWG requires fewer trials, which is preferable in terms of cost- and time-efficiency.

## 3. Conclusions

The Hansen solubility parameter (HSP) is a powerful tool to classify solvents in terms of gelation ability. However, conventional methods can only be used for qualitative evaluation (e.g., solution, opaque or transparent gels, and precipitation). This work aimed to obtain quantitative relationships between HSP and the physical properties of organogels. Twenty-three solvents and 12-hydroxystearic acid (12HSA) were used to prepare organogels, and their properties were evaluated by the critical gelation concentration (CGC), mechanical strength, and light transmittance.

We found correlations between the distance in HSP space (D) and the physical properties of organogels. The most significant correlation was found between D and yield stress ($\tau_{y}$). The gelation mechanism can explain this correlation: as the solubility decreases in a solvent, more 12HSA molecules are used to form SAFiNs. Therefore, the mechanical strength of the organogel increases. Since the solubility of 12HSA is strongly related to D, D and $\tau_{y}$ are strongly correlated as well.

Another significant finding was that the concentration of 12HSA should be defined by the amount of 12HSA molecule in a unit volume of a pure solvent (v) when comparing different solvents. SAFiNs determine the properties of organogels, and the density of fibers in SAFiNs is affected by the number of molecules available in the unit volume. Therefore, if the properties of organogels are different even when v is fixed, it suggests that the difference is attributed to the characteristics of solvents, not the number of 12HSA molecules.

These findings are beneficial when choosing the solvent for gelation. The conventional HSP-based methods require numerous samples to determine the gelation/precipitation boundary because each sample has only binary information: gel or not. In the proposed method, the physical properties of organogels can be a good measure of how close the HSP of solvent is to the boundary. Moreover, the mechanical and optical properties can be tuned by choosing a solvent based on HSP. These insights are expected to facilitate further understanding of supramolecular gels and expand their practical application in numerous fields.

## 4. Materials and Methods

### 4.1. Materials

12-hydroxystearic acid (purity > 80.0%), hexane (purity > 96.0%), heptane (purity > 99.0%), octane (purity > 95.0%), nonane (purity > 98.0%), decane (purity > 99.0%), cyclohexene (purity > 99.0%), and acetone (purity > 99.5%) were purchased from Kanto Chemical Co., Inc., Japan. *o*-xylene (purity > 98.0%), *m*-xylene (purity > 99.0%), *p*-xylene (purity > 99.0%), styrene (purity > 99.0%), *α*-methyl styrene (purity > 99.0%), *n*-butylbenzene (purity > 99.0%), di-isononyl adipate (mixture of branched-chain isomers), *d*-limonene (purity > 95.0%), hexachloroacetone (purity > 98.0%), methyl chloroformate (purity > 96.0%), ethyl chloroformate (purity > 98.0%), fluorobenzene (purity > 99.0%), epichlorohydrin (purity > 99.0%), methylene dichloride (purity > 99.0%), 2-methylfuran (purity > 98.0%), and *γ*-butyrolactone (purity > 95.0%) were purchased from Tokyo Chemical Industry Co., Ltd., Tokyo, Japan. The ultra-pure water was prepared using a Direct-Q Water Purification System supplied by EDM Millipore Corporation, and the resistivity was confirmed to be 18.2 MΩ cm. All chemicals were used as received.

### 4.2. Preparation of Organogels

25.0 mL of solvent was poured into a 50 mL vial, and 12HSA was added by 0.10 to 1.80 wt% with an increment of 0.10 wt%. 12HSA concentration was defined based on three criteria: weight, molar amount, volume. The weight of each component was measured and converted using molar weight and liquid density. The density of liquid was measured by Portable Density Meter (DMA35, Anton Paar GmnH, Austria) at room temperature. The mixture was heated at 70 °C if the boiling point was higher than 75 °C in a thermostat bath (DG401, Yamato Scientific Co., Ltd., Tokyo, Japan). Otherwise, it was heated at 5 °C below its boiling point. The mixture was stirred at 300 rpm at this step using a multi-stirrer (MS-53M, Jeio Tech, Daejeon, Korea). After confirming the complete dissolution of 12HSA, the mixture was cooled at room temperature for 48 h.

### 4.3. Characterization of Gelation Degree

The obtained samples were completely inverted and left for 15 min to separate free liquid by gravity. At this point, if the sample was not gelated, the mixture falls from the container. On the other hand, if the sample is gelated, the sample does not flow and sticks to the container even when it is inverted. The sample weight before and after this gravitational separation was measured to determine the gelation degree defined by Equation (4).

The mechanical properties of 12HSA organogels were measured by HAAKE^TM^ MARS^TM^ 40 Rheometer (Thermo Fisher Scientific Inc., Waltham, MA, USA). The plate and spindle used for the measurements were P35 Ti L and TMP35, respectively (diameter of 35.0 mm). The gel was cut into cylindrical shape using a cork borer, and 1 cm^3^ of the gel was placed on TMP35. The gap between P35 Ti L and TMP35 was set to 0.5 mm, and oscillation stress sweep measurement was conducted with a frequency of 1.0 Hz and at 20 °C. The stress was initially set to 100 Pa and was doubled every 60 s until the storage modulus $G^{\prime }$ of a sample became below 100 Pa. In all measurements, $G^{\prime }$ was greater than 100 Pa if gel is formed and less than 100 Pa after yielding (Figures S2 and S3).

The light transmittance was measured using a light-emitting diode (OS5YKA5111A, OptoSupply Ltd., Fo Tan, Hong Kong, wavelength of 590 nm, luminous intensity of 50 cd) and an optical sensor (NJL7502L, Nisshinbo Micro Devices Inc., San Jose, CA, USA) The sensor and diode were fixed to the wall of the cylinder with a diameter of 34.0 mm. Both were placed on the same diameter and faced each other (Figure S4). To measure the transmittance, the resistivity of the sensor was measured, and the voltage applied on the optical sensor was measured and was converted to light intensity. The light intensity of air (reference of measurement) and the gel was measured in a vial (diameter of 34.0 mm) using these sensor and diode. Using the measured intensity, the light transmittance was calculated from Equation (5).

## Figures and Tables

**Table 1:** List of solvents used in this work with their HSP [30] and CGCs.

Name of Solvent	Abbr.	$\delta_{D}$ [MPa^0.5^]	$\delta_{P}$ [MPa^0.5^]	$\delta_{H}$ [MPa^0.5^]	$w_{CGC}$ [wt%]	$x_{CGC}$ [mol%]	$v_{CGC}$ [mmol/L-Solvent]
Hexane	Al06	29.8	0.0	0.0	0.70	2.40	53.5
Heptane	Al07	30.6	0.0	0.0	0.90	2.65	62.0
Octane	Al08	31.0	0.0	0.0	0.80	2.08	49.6
Nonane	Al09	31.4	0.0	0.0	0.80	1.85	45.1
Decane	Al10	31.4	0.0	0.0	0.70	1.47	36.2
Cyclohexene	Cy06	34.4	1.0	2.0	0.60	2.16	59.5
o-Xylene	CyOx	35.6	1.0	3.1	0.90	2.49	74.9
m-Xylene	CyMx	35.6	1.0	3.1	1.00	2.78	81.7
p-Xylene	CyPx	35.6	1.0	3.1	0.90	2.50	73.5
Styrene	CySt	37.2	1.0	4.1	1.00	2.83	88.0
a-Methyl Styrene	CyMs	37.0	2.4	2.4	0.90	2.26	70.0
n-Butylbenzene	CyBb	34.8	0.1	1.1	0.90	1.99	58.0
Di-Isononyl Adipate	HcDi	33.4	1.8	4.9	1.60	1.21	37.6
d-Limonene	HcLn	34.4	1.8	4.3	0.90	1.96	55.9
Hexachloroacetone	XCa	36.6	3.0	3.0	0.40	0.45	21.7
Methyl Chloroformate	XMc	32.6	9.5	8.5	0.30	0.94	38.5
Ethyl Chloroformate	XEc	32.8	11.0	8.0	0.50	1.37	52.7
Fluorobenzene	XFb	36.2	6.1	2.0	0.89	2.72	95.3
Epichlorohydrin	XEp	35.0	7.6	7.6	0.70	2.23	89.6
Methylene Dichloride	XDc	34.0	7.3	7.1	1.00	3.43	157.4
2-Methylfuran	Mf	34.6	2.8	7.4	0.90	3.20	102.1
Acetone	At	31.0	10.4	7.0	insoluble *	insoluble *	insoluble *
Water	Wt	31.0	16.0	42.3	insoluble *	insoluble *	insoluble *
γ-Butyrolactone	Gb	36.0	16.6	7.4	insoluble *	insoluble *	insoluble *

* Solvents did not dissolve 12HSA under the tested conditions.

**Table 2:** Correlation results ($R^{2}$ and Δ ) between D and various experimental variables.

Correlation of *D* with	$R^{2}[-]$	Δ[-]
$w_{\mathrm{CGC}}$	0.267	0.660
$v_{\mathrm{CGC}}$	0.106	0.648
$x_{\mathrm{CGC}}$	0.046	0.760
$w_{\mathrm{CGC}}$(only alkene and aromatics)	0.195	0.638
$v_{\mathrm{CGC}}$(only alkene and aromatics)	0.650	0.479
$x_{\mathrm{CGC}}$(only alkene and aromatics)	0.261	0.688
$\tau_{y}$(w=1.0 %)	0.037	0.843
$\gamma_{y}$(w=1.0 %)	0.317	0.746
$\alpha_{\mathrm{gel}}$(w=1.0 %)	0.228	0.677
$\tau_{y}$(v=80 mmol/L−solvent)	0.917	0.218
$\gamma_{y}$(v=80 mmol/L−solvent)	0.798	0.376
$\alpha_{\mathrm{gel}}$(v=80 mmol/L−solvent)	0.597	0.485
$\tau_{y}$(x=2.8%)	0.673	0.433
$\gamma_{y}$(x=2.8%)	0.737	0.357
$\alpha_{\mathrm{gel}}$(x=2.8%)	0.676	0.426

**Table 3:** Property of 12HSA organogels obtained at w=1.0 wt%.

Abbr.	$\alpha_{\mathrm{gel}}[-]$	$\tau_{y}[\mathrm{Pa}]$	$\gamma_{y}[-]$
Al06	0.086	1018	0.026
Al07	0.091	1109	0.031
Al08	0.154	972	0.032
Al09	0.157	551	0.030
Al10	0.045	394	0.028
CyOx	0.316	349	0.014
CyMx	0.316	391	0.017
CyPx	0.466	391	0.016
CySt	0.375	303	0.015
CyMs	0.216	562	0.019
CyBb	0.504	371	0.021
HcLn	0.547	403	0.022
XFb	0.111	537	0.013
Mf	0.122	1066	0.020

**Table 4:** Property of 12HSA organogels obtained at v=80 mmol/L−solvent.

Abbr.	$\alpha_{\mathrm{gel}}[-]$	$\tau_{y}[\mathrm{Pa}]$	$\gamma_{y}[-]$
Al06	0.084	615	0.022
Al07	0.106	987	0.031
Al08	0.137	1023	0.034
Al09	0.213	821	0.032
Al10	0.121	724	0.029
Cy06	0.149	659	0.021
CyOx	0.321	354	0.014
CyMx	0.322	397	0.017
CyPx	0.468	408	0.016
CySt	0.375	303	0.015
CyMs	0.320	567	0.021

**Table 5:** Property of 12HSA organogels obtained at x=2.8 mol%.

Abbr.	$\alpha_{\mathrm{gel}}[-]$	$\tau_{y}[\mathrm{Pa}]$	$\gamma_{y}[-]$
Al06	0.066	1095	0.025
Al07	0.075	1199	0.027
Al08	0.121	1032	0.030
Al09	0.173	1060	0.033
Al10	0.257	849	0.029
Cy06	0.141	659	0.021
CyOx	0.359	321	0.014
CyMx	0.316	391	0.017
CyPx	0.456	386	0.017
CyMs	0.243	555	0.019

## Data Availability

Data are contained within the article.

## Supplementary Materials

The following supporting information can be downloaded at: https://www.mdpi.com/article/10.3390/gels9040314/s1, Figure S1: Appearance of prepared gels with 1.0 wt% of 12HSA with various solvents; Figure S2: Storage and loss moduli measured by rheological measurement of prepared gels with 1.0 wt% of 12HSA with various solvents; Figure S3: Stress-strain curve obtained by rheological measurements of prepared gels with 1.0 wt% of 12HSA with various solvents; and Figure S4: Schematic diagram of light transmittance measurement using diode and optical sensor.
