# Supplementary material for: Correlation between Physical Properties of 12-Hydroxystearic Acid Organogels and Hansen Solubility Parameters
Source: Gels. 2023 Apr 7;9(4):314. doi: 10.3390/gels9040314 (PMC10137447; doi:10.3390/gels9040314)
Supplement: Supplementary file 1 [file gels-09-00314-s001.zip › gels-2277964-supplementary.pdf]

gels

MDPI

Article

Correlation between Physical Properties of 12-Hydroxystearic Acid Organogels and Hansen Solubility Parameters

Yuya Murakami [1,*], Taisei Uchiyama [2] and Atsushi Shono [1]

[1] Department of Industrial Chemistry, Faculty of Engineering, Tokyo University of Science, 6-3-1, Nijuku, Katsushika-ku, Tokyo 125-8585, Japan
[2] Institute of Innovative Research, Tokyo Institute of Technology, 2-12-1, Ookayama, Meguro-ku, Tokyo 152-8550, Japan
* Correspondence: y.murakami@ci.tus.ac.jp; Tel.: +81-3-5876-1717

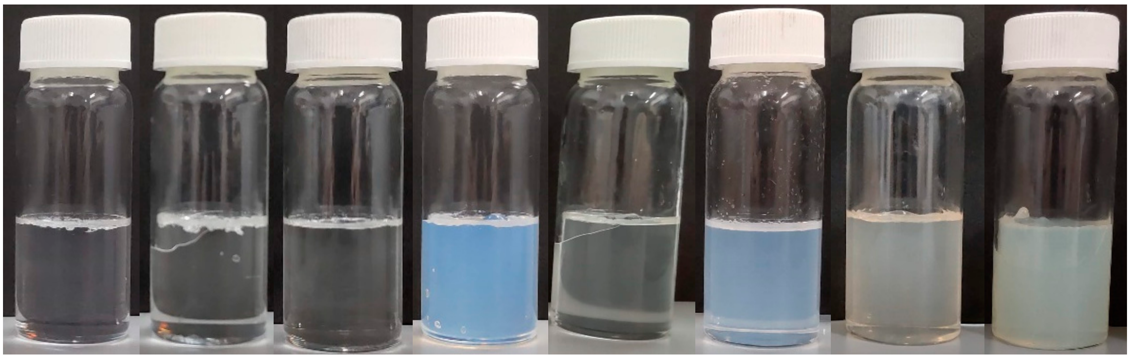

Figure S1. Appearance of prepared gels with 1.0 wt% of 12HSA with various solvents (diameter of vial is 34.0 mm): (a) o-Xylene, (b) m-Xylene, (c) p-Xylene, (d) Styrene, (e) n-Butylbenzene, (f) Di-Isononyl Adipate, (g) Fluorobenzene, (h) 2-Methylfuran.

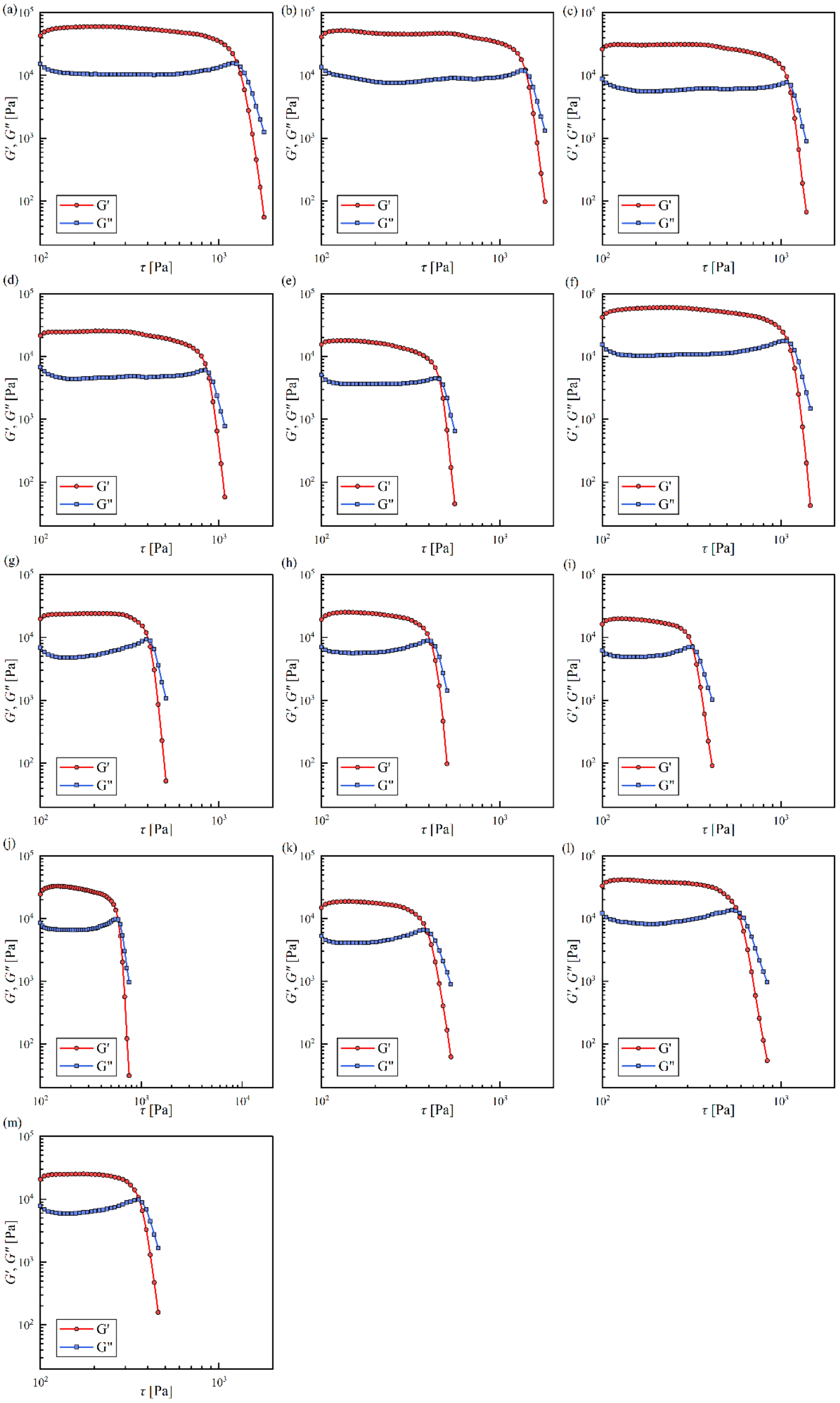

Figure S2. Storage and loss moduli measured by rheological measurement of prepared gels with 1.0 wt% of 12HSA with various solvents: (a) Hexane, (b) Heptane, (c) Octane, (d) Nonane, (e) Decane, (f) o-Xylene, (g) m-Xylene, (h) p-Xylene, (i) Styrene, (j) a-Methyl Styrene, (k) n-Butylbenzene, (l) Fulorobenzene, (m) 2-Methylfuran.

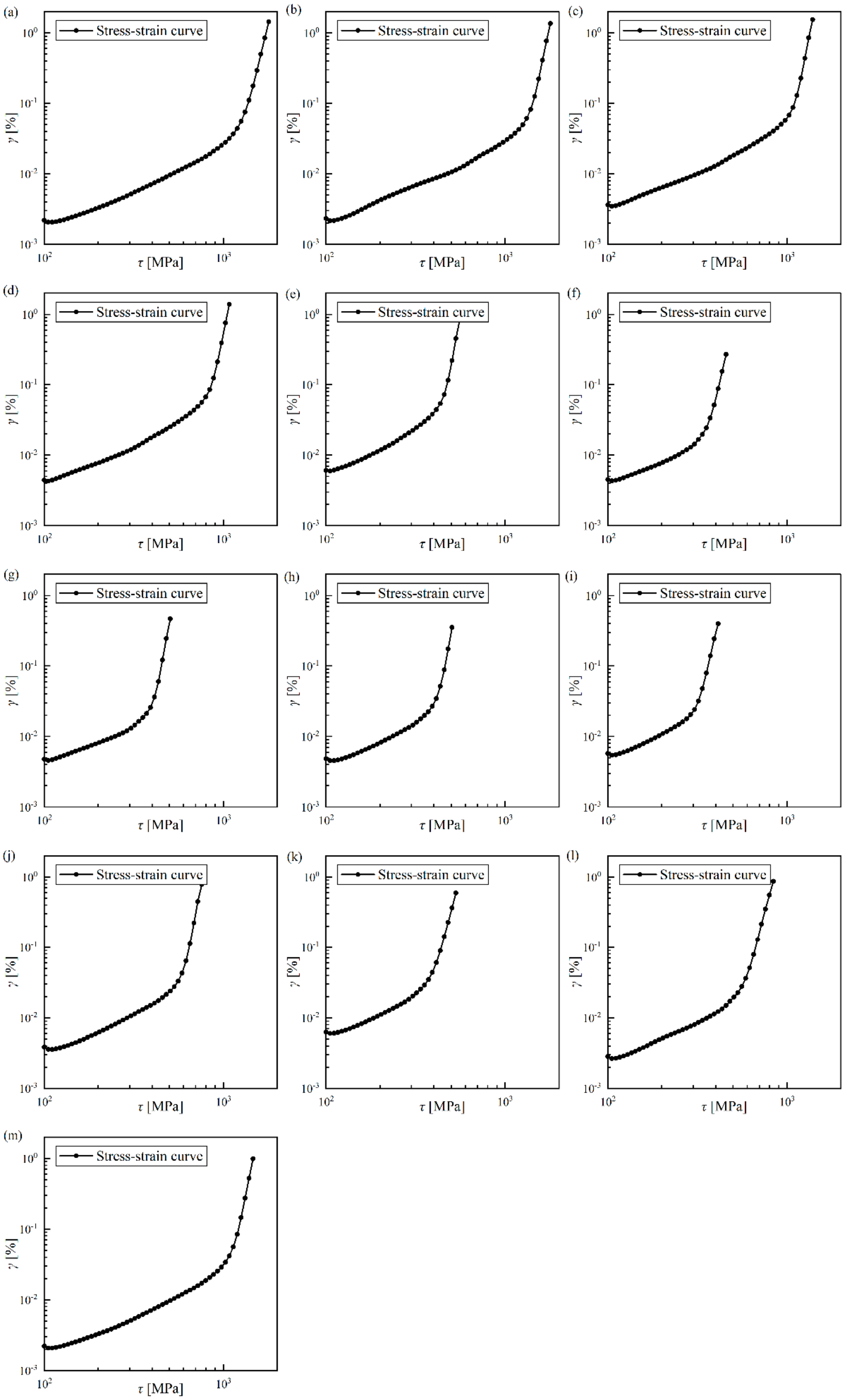

Figure S3. Stress-strain curve obtained by rheological measurements of prepared gels with 1.0 wt% of 12HSA with various solvents: (a) Hexane, (b) Heptane, (c) Octane, (d) Nonane, (e) Decane, (f) o-Xylene, (g) m-Xylene, (h) p-Xylene, (i) Styrene, (j) a-Methyl Styrene, (k) n-Butylbenzene, (l) Fuloro-benzene, (m) 2-Methylfuran.

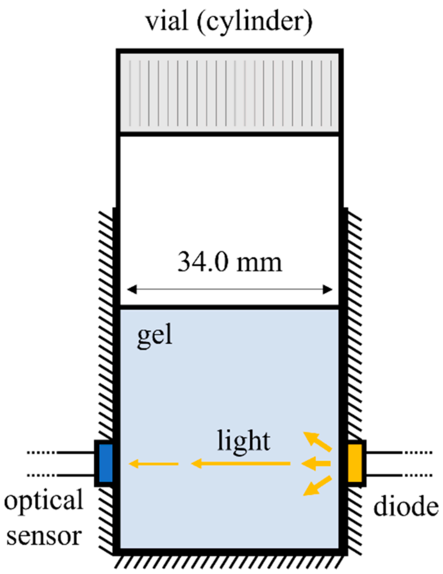

Figure S4. Schematic diagram of light transmittance measurement using diode and optical sensor.
